# NFIL3 Acts as a Nuclear Factor to Increase Osteosarcoma Progression

**DOI:** 10.1155/2019/4068521

**Published:** 2019-11-30

**Authors:** Jiaxing Xu, Gongping Xu, Tingxin Zhang, Tiantian Chen, Wei Zhao, Guangyou Wang

**Affiliations:** ^1^Department of Surgery, The First Affiliated Hospital of Harbin Medical University, Harbin 150000, China; ^2^Department of Orthopedics, The Second Affiliated Hospital of Harbin Medical University, Harbin 150081, China; ^3^Department of Neurobiology, Harbin Medical University, Harbin 150081, China

## Abstract

**Purpose:**

Osteosarcoma is one of the most common primary malignant, aggressive bone neoplasms. However, the mechanisms of osteosarcoma proliferation, migration, and invasion are not well understood. To explore the possible mechanism of osteosarcoma progression, we used a public database for gene analysis to identify the possible factors that are important in osteosarcoma progression. Nuclear factor interleukin 3 (NFIL3) regulated was highly expressed in sarcoma tissues. In this study, we meant to probe the function of NFIL3 in osteosarcoma proliferation, migration, and invasion.

**Methods:**

The expression of NFIL3 in osteosarcoma tissues was analysed via RT-PCR and immunohistochemistry staining. In order to elucidate the function of NFIL3 in osteosarcoma, we performed cell growth assays and colony formation assays to explore the role of NFIL3 in proliferation in osteosarcoma cells. Futhermore, we analysed osteosarcoma cell migration and invasion via wound healing assays and transwell migration and invasion assays.

**Results:**

NFIL3 is overexpressed in osteosarcoma tissues; 15 of the 20 osteosarcoma tissues analysed highly expressed NFIL3. Our in vitro experiments confirmed that NFIL3 promoted the proliferation of M6-63 and SaOS2 cells (*P* < 0.01). In addition, NFIL3 promoted the migration and invasion of osteosarcoma cells (*P* < 0.05).

**Conclusion:**

NFIL3 is highly expressed in osteosarcoma tissues and thus promotes the proliferation, migration, and invasion of osteosarcoma cells. NFIL3 is potential to become a new target for development of novel treatment strategies of osteosarcoma.

## 1. Introduction

Osteosarcoma is one of the most frequent malignant bone tumours, and it is most prevalent in children and young adults [1], accounting for 20% of primary bone cancers; approximately 50% of tumours are found in the proximal tibial and distal femoral areas [2]. Osteosarcoma is highly aggressive [3, 4], with rapid growth and early metastasis. Patients with lung metastatic disease have a worse prognosis with a five-year survival rate of approximately 20% at the time of diagnosis [5–7]. Currently, surgical treatment combined with preoperative and postoperative chemotherapy is the main treatment for osteosarcoma. However, even after the combination of traditional surgical treatment and neoadjuvant chemotherapy, there are still remaining problems such as reduced patient quality of life and tumour recurrence. Hence, it is urgent to elucidate the underlying mechanisms of osteosarcoma progression.

NFIL3 (nuclear factor interleukin 3 regulated) was first reported as an interleukin 3- (IL3-) induced nuclear factor in pro-B lymphocytes [8, 9]. It is a basic leucine zipper transcription factor in the immune system that is also known as E4BP4 (E4-binding protein 4) and is widely expressed in human tissues. NFIL3 is indispensable in the early period of cNK cells [10–12] and simultaneously inhibits apoptosis in numerous cell types, from B-cells to motor neurons, in which 50% undergo apoptosis [13]. In recent years, several studies have shown that NFIL3 mediates chemotherapy resistance in choriocarcinoma cells [14] and is often overexpressed in cancers with a poor prognosis [15].

In this study, we obtained the gene expression profile matrix and clinicopathological information of sarcoma patients from a database (http://gepia.cancer-pku.cn/detail.php?Gene=NFIL3) and analysed the expression of NFIL3 in sarcoma and matched normal tissues. After a series of studies, we provide the first evidence that the expression levels of NFIL3 are consistently increased in osteosarcoma tissues. NFIL3 promotes cell proliferation, invasion, and migration of osteosarcoma cells ex vivo. This is a novel phenomenon that suggests NFIL3 has potential to become a novel target for the immunotherapy and targeted therapy of osteosarcoma which will be a new direction for our future research.

## 2. Materials and Methods

### 2.1. Clinical Samples

Twenty pairs of pretherapeutic primary osteosarcoma and matched adjacent biopsy samples were obtained from Liaoning Provincial Hospital. The written informed consents to participate in this study were obtained from the corresponding patients, and the Ethics Committee of Liaoning Provincial Hospital approved this work.

### 2.2. Cell Culture

The human osteosarcoma cell lines MG-63 and SAOS2 were obtained from the Cell Bank of the Chinese Academy of Sciences (Shanghai, China). DMEM (Gibco, USA) supplemented with 10% FBS (Gibco) and 1% penicillin-streptomycin (10 U/ml) was used to culture the cells at 37°C in a 5% CO_2_ atmosphere.

### 2.3. Total RNA Extraction and Quantitative Real-Time RT-PCR

Total RNA was extracted from clinical tissue using Trizol reagent (Invitrogen). Total RNA was then reversely transcripted into cDNA with the TransScript® One-Step gDNA Removal and cDNA Synthesis SuperMix Kit (Trans®, China). An ABI 7900 RT-PCR system and TransScript® Tip Green qPCR SuperMix Kit (Trans®) were used to perform qRT-PCR according to the manufacturer's instructions. The primers were purchased from GenePharma (Shanghai, China).

### 2.4. Transfection

pLVX-puro lentiviral vectors were transfected to construct NFIL3 overexpressing osteosarcoma cell lines. Reverse transcription PCR was performed to obtain and amplify the full-length sequence of NFIL3, and the digested sequence of NFIL3 was inserted into the pLVX vector. pLKO-GFP-shRNA lentiviral vectors were used to knockdown NFIL3 in osteosarcoma cells. The specific primers were as follows: NFIL3 RT-PCR primer (F: TGACCGAGGCTCTTACAC, R: GGAGGATCGGTTGACTTG), NFIL3 overexpression primer (F: CTCGAGAGGAGCCTGACGGATTTA, R: GGATCCCAGCCTTCGCATGGACTA), NFIL3-sh1 (sense: CCACACAAGCTCCGGATCAAA, antisense: TTTGATCCGGAGCTTGTGTGG), and NFIL3-sh2 (sense: CCAGAGAACTTGTATTTGAAG, antisense: CTTCAAATACAAGTTCTCTGG). To get packaged lentiviral particles, the plasmids accompanied with packaging plasmids (pSPAX2 and pMD.2G) were transfected into HEK-293TN cells for 48 h to obtain pseudo lentiviral particles. Aiming to construct stable lentivirus transduction cell lines, 10 MOI GFP lentiviral particles were employed to treat with osteosarcoma cells for at least 12 h, and then flow cytometry was used to sort GFP-positive cells after obtaining GFP-positive cells which would be further cultured for 72 h.

### 2.5. Immunohistochemistry Staining

Sections (5 mm) of the tissue microarray were mounted on poly-L-lysine (Sigma, St. Louis, MO, USA) coated slides and prepared for immunohistochemistry (IHC) after dewaxing and dehydrating. Antigen retrieval treatment was performed using citrate buffer in a microwave, and the slides were then cooled for 20–30 min. After washing with 0.01 M Tris-buffered saline (pH 7.4), the sections were incubated with 1% BSA followed by overnight incubation at 4°C with a primary antihuman antibody against NFIL3 (Proteintech, China) at a dilution of 1 : 100; then, the slides were incubated with an HRP-conjugated secondary detection antibody and DAB. Each experiment was also carried out according to the instructions but with PBS instead of the primary antibody as a negative control and with a verified positive biopsy as a positive control. NFIL3 was expressed in the nuclei of cells. The expression was visualized as yellow or brown granules in fine particles. The IHC results for all cases were scored using the Axiotis pathological score standard. In brief, protein expression was semiquantified according to the cell staining intensity and the percentage (ranging from 0 to 100%) of positively stained cells of any intensity, with scores ranging from 0 to 3 as follows: 0–5% = 0 (−); 6–30% = 1 (+); 31–50% = 2 (++); and ≥51% = 3 (+++). Photographs were taken with a Leica digital imaging system (200×, bar = 50 *μ*m).

### 2.6. Cell Growth Assay

A total of 1 × 105 human osteosarcoma MG-63 and SAOS2 cells were incubated on the bottom of a 6-well plate with 2000 *μ*L serum medium and incubated at 37°C in 5% CO_2_. The cells were counted every 24 h using a Countstar IC1000 (Countstar, USA). All experiments were performed in triplicate and repeated three times.

### 2.7. Colony Formation Assay

In brief, after 48 h of transfection, 1 × 103 human osteosarcoma MG-63 and SAOS2 cells were initially incubated into each well of a 6-well plate and maintained in a medium containing 10% FBS, and the medium was changed every two days. Colonies were observed with naked eyes when the cells had incubated for 12 days at 37°C in 5% CO_2_. A 1% crystal violet solution was used to stain the colonies for 30 min before being counted by ImageJ software. All experiments were repeated three times.

### 2.8. Wound Healing Assay

Human osteosarcoma MG-63 and SAOS2 cells were inoculated in a 6-well plate and cultured with 10% complete DMEM. When the cells covered plates, a straight line was drawn in each well with a 200-*μ*l pipette tip, and the unattached cells were washed away with PBS. The culture medium was substituted by 5% complete DMEM, and then the cells were observed and photographed every 24 h.

### 2.9. Migration and Invasion Assays

Transwell chambers (Coster, USA) were used to perform the migration and invasion assays. A total of 1 × 105 cells in 200 *μ*L serum-free DMEM were incubated in the upper chamber. 800 *μ*L complete DMEM with 10% FBS was used to fufill the lower chambers. After seeding for 48 h at 37°C, the cells on the upper side of the chamber were gently swiped off with cotton swabs. The invaded cells on the lower side of the chamber were fixed with a 1% crystal violet solution 30 min before being counted. The Olympus inverted microscope (200×) was employed to take the photographs. The migration and invasion cell numbers were counted using ImageJ software.

### 2.10. Statistical Analysis

All statistical analyses were performed using SPSS 19 software (SPSS, USA). One-way ANOVA was used to anlayse the significance of the differences among groups. All the results are reported as mean ± SD. *P* < 0.05 and *P* < 0.01 were regarded statistically significant.

## 3. Results

### 3.1. NFIL3 is Highly Expressed in Osteosarcoma

**Figure 1:**
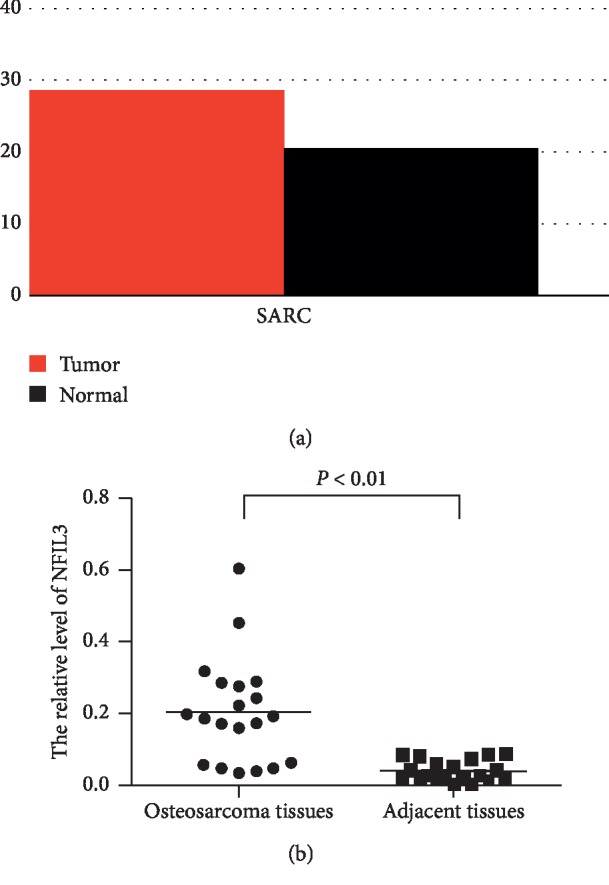
NFIL3 is highly expressed in human osteosarcoma tissues. (a) The gene expression of NFIL3 is higher in 262 cases of sarcoma tissues than in 2 cases of normal tissues according to a public database analysis (http://gepia.cancer-pku.cn/detail.php?gene=NFIL3) and the gene expression profile across all tumour samples and paired normal tissues. The height of the bar represents the median expression of certain tumour type or normal tissue (tumour vs. normal: 28.54 vs. 20.61). (b) NFIL3 expression is higher in osteosarcoma tissues than in adjacent nontumour tissues according to qRT-PCR. (*P* < 0.01).

The expression level of NFIL3 in sarcoma tissue and normal bone tissues was compared through public database analysis, and NFIL3 was highly expressed in sarcoma tissues (Figure 1(a)). To further confirm whether NFIL3 was highly expressed in osteosarcoma tissues, the expression levels of NFIL3 in 20 pairs of osteosarcoma tissues and matched nontumour samples were quantified by quantitative real-time PCR. The expression level of NFIL3 was higher in osteosarcoma tissues than in matched nontumour tissues (Figure 1(b)).

**Figure 2:**
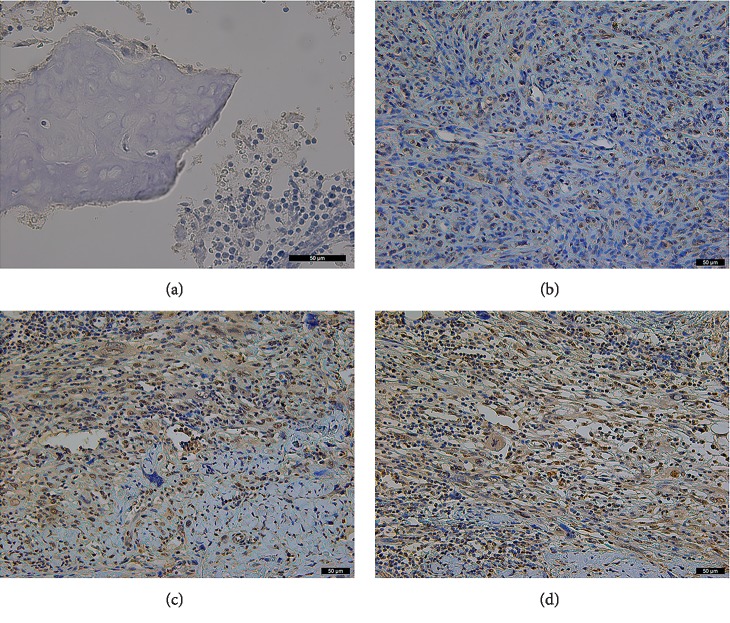
NFIL3 is expressed in human osteosarcoma tissue and adjacent nontumour tissue (immunohistochemistry staining). (a) NFIL3 is negative expressed in adjacent nontumour tissue. (b) NFIL3 is moderate expressed in human osteosarcoma tissue. (c and d) NFIL3 is highly expressed in human osteosarcoma tissues. (400×, bar = 50 *μ*m).

Immunohistochemical staining analysis was used to further confirm this result, and 15 of 20 osteosarcoma tissues highly expressed NFIL3 (Figures 2(c) and 2(d)).

These results indicated that NFIL3 is highly expressed in osteosarcoma tissues.

### 3.2. NFIL3 Promotes the Proliferation of M6-63 and SaOS2 Cells

**Figure 3:**
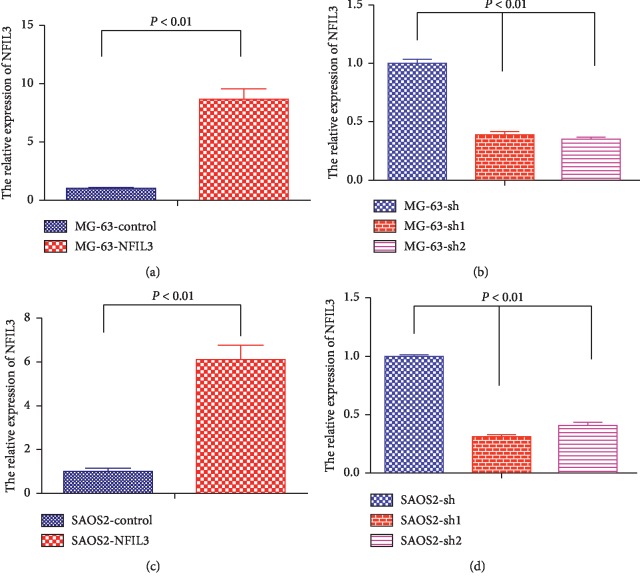
The relative expression of NFIL3 in human osteosarcoma cells (qRT-PCR). (a) Lentivector-NFIL3 increased the expression of NFIL3 compared with the control group in MG-63 cells. (b) shRNA-lentivector-NFIL3 decreased expression of NFIL3 compared with the NC group in MG-63 cells. (c) Lentivector-NFIL3 increased the expression of NFIL3 compared with the control group in SAOS2 cells. (d) shRNA-lentivector-NFIL3 decreased expression of NFIL3 compared with the NC group in SaOS2 cells (*P* < 0.01).

**Figure 4:**
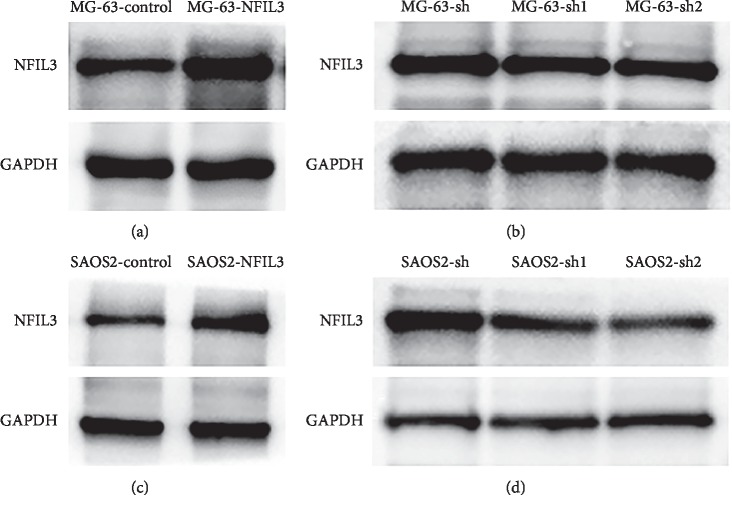
The relative expression of NFIL3 in human osteosarcoma cells (western blot). (a) Lentivector-NFIL3 increased the expression of NFIL3 compared with the control group in MG-63 cells. (b) shRNA-lentivector-NFIL3 decreased expression of NFIL3 compared with the NC group in MG-63 cells. (c) Lentivector-NFIL3 increased the expression of NFIL3 compared with the control group in SaOS2 cells. (d) shRNA-lentivector-NFIL3 decreased expression of NFIL3 compared with the NC group in SaOS2 cells.

To confirm whether NFIL3 affects the proliferation of osteosarcoma cells in vitro, lentiviral vectors were employed to overexpress and knockdown NFIL3 in MG-63 and SaOS2 cells (Figures 3 and 4).

**Figure 5:**
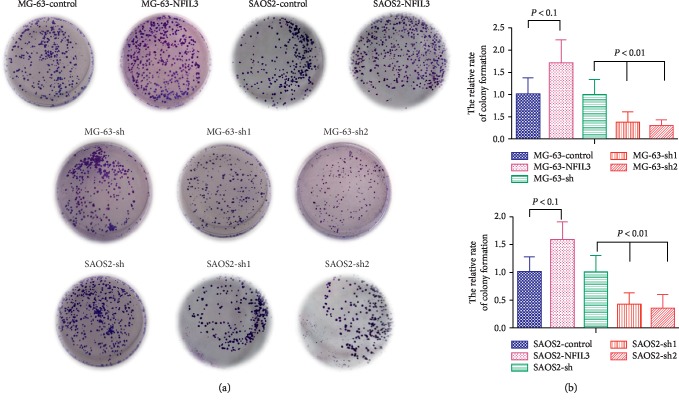
NFIL3 increases the colony formation rate of human osteosarcoma cells. (a-b) NFIL3 overexpression increased the colony formation rate of MG-63 and SaOS2 cells, and NFIL3 knockdown decreased the colony formation rate of MG-63 and SaOS2 cells (*P* < 0.01).

Our colony formation assays demonstrated that forced NFIL3 expression in MG-63 and SaOS2 cells promoted cell proliferation compared with the control conditions, whereas NFIL3 knockdown obviously restricted osteosarcoma cell proliferation in these two cell lines compared with the control conditions, as shown in Figure 5(a), and Figure 5(b) is the statistical quantification of Figure 5(a) (*P* < 0.01).

**Figure 6:**
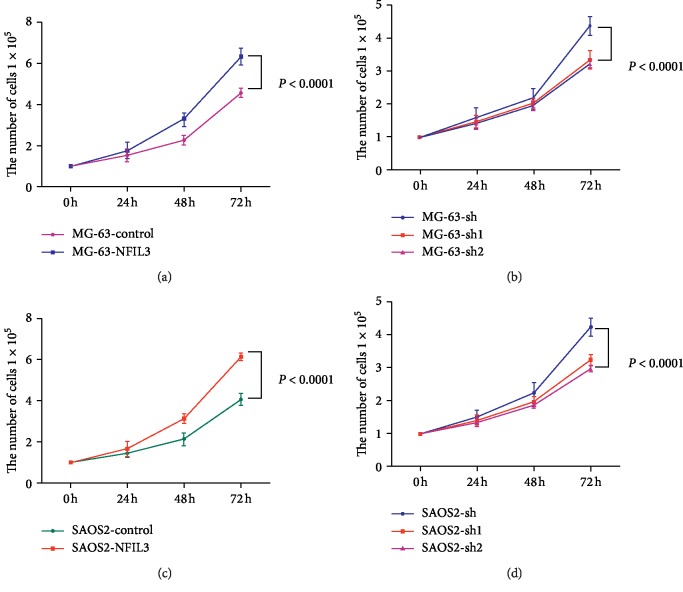
NFIL3 increases the proliferation of human osteosarcoma cells. (a) NFIL3 overexpression increased the proliferation of MG-63 cells. (b) NFIL3 knockdown decreased the proliferation of MG-63 cells. (c) NFIL3 overexpression increased the proliferation of SaOS2 cells. (d) NFIL3 knockdown decreased the proliferation of SaOS2 cells (*P* < 0.01).

To confirm these results, a cell growth curve was generated. Overexpression of NFIL3 increased the proliferation of MG-63 and SaOS2 cells (Figures 6(a) and 6(c), *P* < 0.01), whereas knockdown of NFIL3 decreased the proliferation of MG-63 and SaOS2 cells (Figures 6(b) and 6(d), *P* < 0.01).

These results indicated that NFIL3 increased the proliferation of osteosarcoma cells.

### 3.3. NFIL3 Promotes the Migration and Invasion of Osteosarcoma Cells

**Figure 7:**
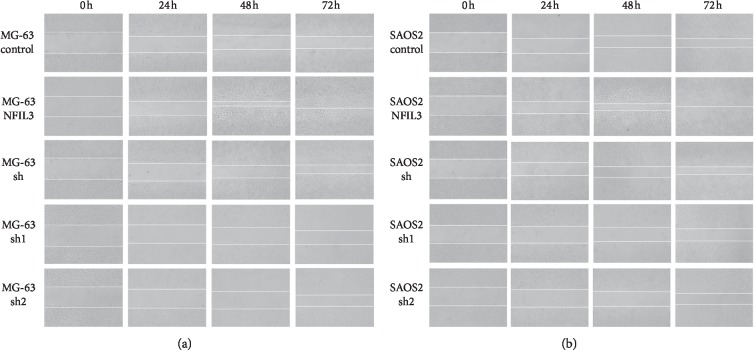
NFIL3 increases the migration of human osteosarcoma cells (wound healing assay). (a) NFIL3 overexpression increased the migration of MG-63 cells, and NFIL3 knockdown decreased the migration of MG-63 cells. (b) NFIL3 overexpression increased the migration of SAOS2 cells, and NFIL3 knockdown decreased the migration of SAOS2 cells.

We performed wound healing assays using MG-63 and SaOS2 cells and observed them every 24 hours. Overexpression of NFIL3 greatly enhanced the migration of MG-63 and SaOS2 cells, whereas knockdown of NFIL3 decreased the migration of M6-63 and SaOS2 cells (Figures 7(a) and 7(b)).

**Figure 8:**
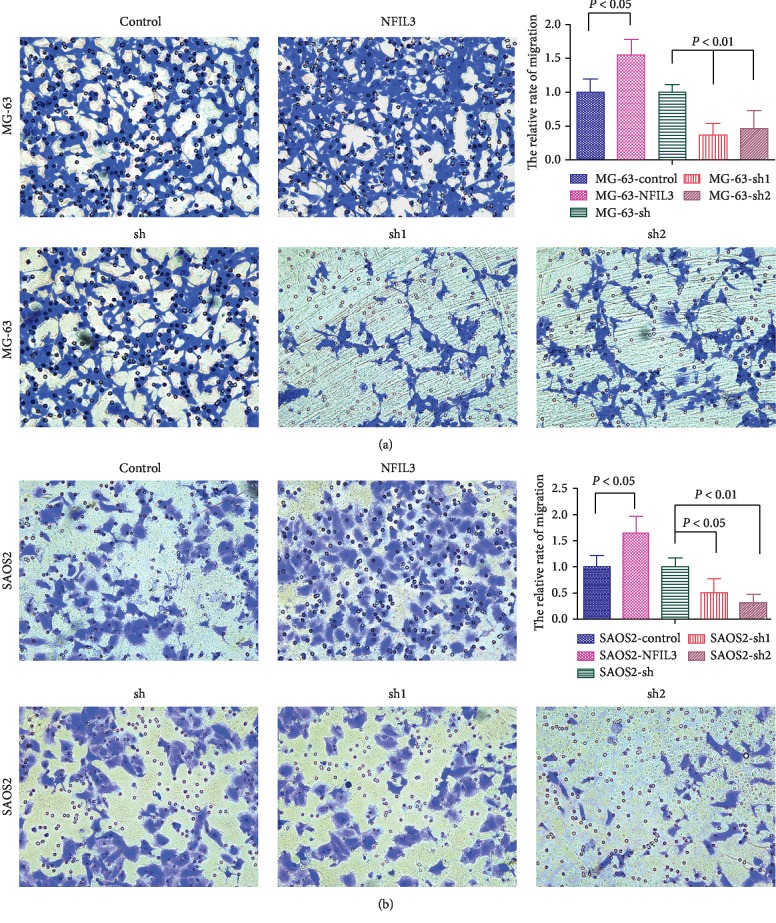
NFIL3 increases the migration of human osteosarcoma cells (transwell migration assay). (a) Overexpressed NFIL3 enhanced the migration of MG-63 cells, and NFIL3 knockdown restricted the migration of MG-63 cells. (b) NFIL3 overexpression increased the migration of SaOS2 cells, and NFIL3 knockdown decreased the migration of SAOS2 cells (*P* < 0.05).

**Figure 9:**
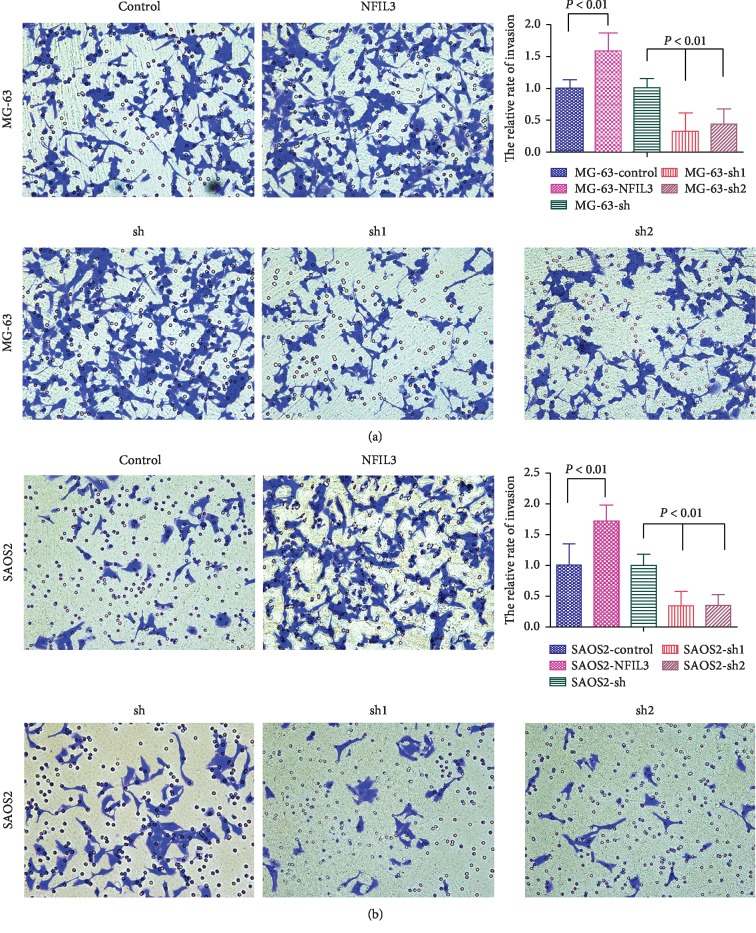
NFIL3 increases the invasion of human osteosarcoma cells (transwell invasion assay). (a) NFIL3 overexpression increased the invasion of MG-63 cells, and NFIL3 knockdown decreased the invasion of MG-63 cells. (b) NFIL3 overexpression increased the invasion of SaOS2 cells, and NFIL3 knockdown decreased the invasion of SaOS2 cells (*P* < 0.01).

Transwell migration and invasion assays showed that overexpressed NFIL3 significantly promoted migration and invasion of osteosarcoma cells, whereas knockdown of NFIL3 noticeably inhibited the migration and invasion of osteosarcoma cells (Figures 8 and 9, *P* < 0.05).

The wound healing assays and transwell migration and invasion assays showed that NFIL3 acts as an oncogene and boosts the migration and invasion of osteosarcoma cells.

## 4. Discussion

Immunotherapy refers to the enhancement of the host immune response through artificial stimulation and increased cytotoxicity and secondary enhancement of the body's antitumour immune response, cancer tissue destruction, and tumour cell apoptosis promotion [16]. As early as 1891, scientists discovered that bacterial extraction and treatment with these substances could affect the proliferation of osteosarcoma [17]. With an in-depth understanding of the immune mechanism, more immunotherapy targets for osteosarcoma have been found, but the related mechanisms are still not fully understood [18–21]. Studies have shown that in addition to directly removing tumour cells, targeted therapy can also participate in immune regulation and accelerate the initiation of tumour-specific cytotoxic T lymphocytes (CTLs) [22], enhance immune regulation, and continuously kill target cells with high efficiency. Immunotherapy combined with targeted therapy may have synergistic mechanisms that amplify cytotoxic effects, inhibit drug-resistant tumour cell formation [23], relieve apoptosis inhibition, and improve the tumour microenvironment.

NFIL3 takes part in a number of immune processes, and is a key factor in the early development of cNK cells [10, 12, 24]. In 2011, Smith et al. found that NFIL3 is involved in the induction of anti-inflammatory effects mediated by STAT3 [25]. In 2013, Keniry et al. demonstrated that NFIL3 is overexpressed in different cancers and that it could alter cancer cell behaviour and FOXO function, thereby influencing therapeutic effects [15]. In 2017, Peng et al. found that chemotherapy resistance in choriocarcinoma cells may be caused by STAT3/NFIL3 pathway activation [26]. Our research group collected and analysed information from a bioinformatics database. It was found that NFIL3 was highly expressed in human osteosarcoma tissues and adjacent tissues. Moreover, immunohistochemical detection also confirmed this finding. Therefore, we assume that NFIL3 is closely associated with the morbidity, development, and prognosis of osteosarcoma. To further study the relationship of NFIL3 with osteosarcoma, we performed cytological experiments, including RT-PCR, wound healing assays, migration and invasion assays, cell cloning assays, cell proliferation assays, and other assays. It was found that overexpression of NFIL3 promoted the proliferation, migration, and invasion of osteosarcoma cell line MG-63 and SaOS2. When the NFIL3 gene was knocked out, the proliferation ability, invasion ability, and metastasis ability of osteosarcoma cells were significantly inhibited.

For the first time, we reveal the phenomenon of high NFIL3 expression in osteosarcoma and peritumoural tissue and by immunohistochemical and cytological experiments, further confirmed that NFIL3 is a crucial factor in the occurrence, development, and metastasis of osteosarcoma. We believe that NFIL3 will be a novel target for development of treatment strategy of osteosarcoma; the underlying mechanism was not further elucidated in this article, but it will be determined in future studies.

## 5. Conclusion

NFIL3 is highly expressed in osteosarcoma tissues, thus promoting the proliferation, migration, and invasion of osteosarcoma cells. NFIL3 may become a new target for the treatment of osteosarcoma.

## Data Availability

The detailed data used to support the findings of this study are available from the corresponding author upon request.
